# Severity and Diurnal Improvement of Morning Stiffness Independently Associate with Tenosynovitis in Patients with Rheumatoid Arthritis

**DOI:** 10.1371/journal.pone.0166616

**Published:** 2016-11-16

**Authors:** Yoshihisa Kobayashi, Kei Ikeda, Takayuki Nakamura, Mieko Yamagata, Takuya Nakazawa, Shigeru Tanaka, Shunsuke Furuta, Takeshi Umibe, Hiroshi Nakajima

**Affiliations:** 1 Department of Allergy and Clinical Immunology, Chiba University Hospital, Chiba, Japan; 2 Rheumatology Center, Matsudo City Hospital, Matsudo, Chiba, Japan; 3 Department of Internal Medicine, Chiba Aoba Municipal Hospital, Chiba, Japan; 4 Research Center for Allergy and Clinical Immunology, Asahi General Hospital, Asahi, Chiba, Japan; 5 Department of Rheumatology, National Hospital Organization Shimoshizu Hospital, Yotsukaido, Chiba, Japan; 6 Department of Rheumatology, Allergy, and Clinical Immunology, National Hospital Organization Chiba-East Hospital, Chiba, Japan; Professor, JAPAN

## Abstract

**Background and objectives:**

Although morning stiffness has long been recognized as a characteristic feature of rheumatoid arthritis (RA), it is no more included in the 2010 ACR/EULAR Classification Criteria or in the current major instruments for evaluating disease activity of RA. In this cross-sectional study, we aimed to determine the independent value and the optimal measurement of morning stiffness by clarifying the associations between morning stiffness and synovial inflammation.

**Patients and methods:**

We enrolled 76 consecutive RA patients who underwent musculoskeletal ultrasound examination and agreed to participate in the study. In addition to asking the duration of morning stiffness, we asked patients to complete a diagram which represents the time course of their morning stiffness in the dominant hand. Based on this diagram, we calculated the severity and the diurnal improvement of morning stiffness. We also determined the activity of intra-articular synovitis in 11 joints and tenosynovitis in 8 tendons/tendon compartments in the same hand by using power Doppler (PD) ultrasound with a semiquantitative score (0–3).

**Results:**

For intra-articular synovitis, swollen/tender joint counts more strongly correlated with total PD scores (ρ = 0.379–0.561, p ≤ 0.001) than did any parameters of morning stiffness (ρ = 0.217–0.314, p = 0.006–0.021). For tenosynovitis, however, the severity on awakening and the improvement of morning stiffness more strongly correlated with total PD scores (ρ = 0.503–0.561, p < 0.001) than did swollen/tender joint counts (ρ = 0.276–0.388, p = 0.001–0.016). Multivariate analyses identified the severity on awakening and the improvement but not the duration of morning stiffness as factors that independently associate with the total tenosynovial PD score.

**Conclusions:**

Our data demonstrate a pathophysiological link between morning stiffness and tenosynovitis and also give an insight into the optimal measurement of morning stiffness. Our data support an independent value of evaluating morning stiffness in the management of RA.

## Introduction

Morning stiffness has long been recognized by both patients and rheumatologists as a characteristic feature of rheumatoid arthritis (RA) [1] and was thus included in ACR 1981 remission criteria [2] and 1987 diagnostic criteria for RA [3]. Thereafter, morning stiffness has been widely used in decision-making on changing medication in daily practice [4, 5] and in clinical trials [6, 7]. Studies have also shown that stiffness has considerable impact on patients’ daily life and work [8–11].

Nevertheless, morning stiffness is no more included in 2010 ACR/EULAR Classification Criteria for RA [12, 13] or in the current major instruments for evaluating disease activity of RA such as Disease Activity Score (DAS) [14–16], ACR Core Set [17], Simplified Disease Activity Index (SDAI) [18], and 2011 ACR/EULAR Provisional Definition of Remission [19]. Some studies which these instruments/definitions are based on failed to demonstrate an independent value of assessing morning stiffness [14, 20].

Most of the previous studies measured the duration of morning stiffness [6, 21], while there is conflicting evidence on the optimal measurement of morning stiffness (e.g. duration vs. severity) [22]. In addition, although the typical morning stiffness with inflammatory disorders is considered to improve with activities [23], this possibly important feature of morning stiffness is captured neither by duration nor severity. Furthermore, the lack of an objective, external comparator that represents disease activity, such as imaging findings, was also a major limitation of the previous studies.

In this study, we captured the time course of morning stiffness by employing a patient-reported diagram. We also determined the activity of intra-articular synovitis and tenosynovitis by using musculoskeletal ultrasound, which has been reported to be more accurate than clinical information [24–26], aiming to elucidate the associations between morning stiffness and synovial inflammation.

## Methods

### Patients

We recruited consecutive patients who fulfilled the 2010 ACR/EULAR Classification Criteria for RA [12, 13] and underwent musculoskeletal ultrasound in our hospitals from April 2014 to March 2015. Patients receiving corticosteroids ≥ 10 mg/day were excluded.

The study was approved by Ethics Committee of Chiba University and patients’ written informed consent was obtained according to Declaration of Helsinki.

### Clinical and laboratory assessment

We collected information on patients’ characteristics and current treatment from medical records. We performed clinical evaluation of disease activity in the morning (8:30–12:00) and calculated DAS28 (C-reactive protein-based) [15] and SDAI [18].

### Assessment of morning stiffness

**Fig 1:**
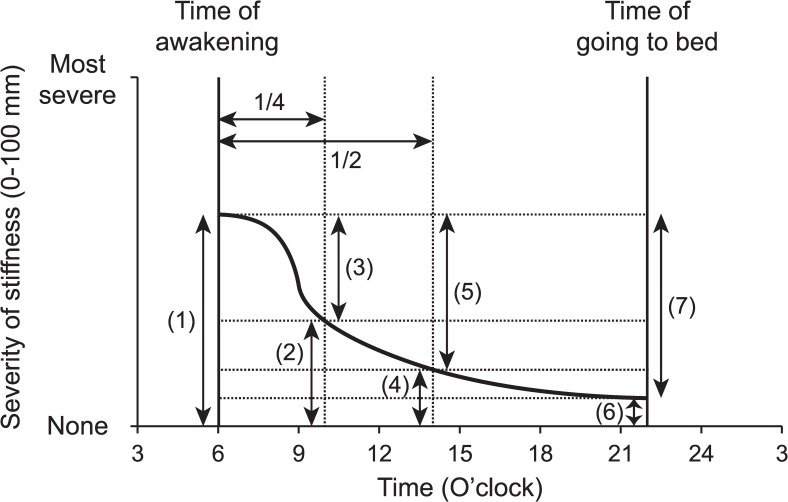
An example diagram for time course of morning stiffness in dominant hand. (1) Severity of stiffness on awakening, (2) Severity of stiffness at the first 1/4 of total awake time, (3) Improvement of stiffness at the first 1/4 of total awake time, (4) Severity of stiffness at the first 1/2 of total awake time, (5) Improvement of stiffness at the first 1/2 of total awake time, (6) Severity of stiffness at bed time, (7) Improvement of stiffness at bed time.

We asked patients about their representative morning stiffness during the past week in their hand with stronger arthritic symptoms. In addition to the duration (i.e. time until maximum improvement [22]), we asked patients to report the severity and time course of their morning stiffness using a diagram (Fig 1). Patients were first asked to place vertical lines which indicated their representative times of awakening and going to bed, respectively. Patients were next asked to indicate the severity of stiffness on wakening based on the visual analogue scale (VAS) (0–100 mm) and draw a free curved line which demonstrates the time course of stiffness during awake time.

Based on this diagram, we determined the severity of stiffness on awakening, at the first 1/4 of total awake time, at the first 1/2 of total awake time, and at bed time. We also determined the diurnal improvement of morning stiffness at these time points by subtraction (Fig 1).

### Musculoskeletal ultrasound

Ultrasound was performed within an hour after clinical and laboratory evaluation in an air-conditioned room (temperature 24–26°C) by rheumatologists trained for musculoskeletal ultrasound, who were blinded to the clinical/laboratory information. Gray-scale (GS) and power Doppler (PD) ultrasound examination of dominant hand was performed using either a HI VISION Avius, a HI VISION Ascendus (Hitachi Medical Corporation, Tokyo, Japan), or an Aplio XG (Toshiba Medical Systems Corporation, Tochigi, Japan), depending on availability. For intra-articular synovitis, 11 joints (1^st^ interphalangeal and 2^nd^-5^th^ proximal interphalangeal joints, 1^st^-5^th^ metacarpophalangeal joints, and wrist [radiocarpal/midcarpal/distal radioulnar joints]) were scanned from the dorsal aspect. For tenosynovitis, 8 tendons/tendon compartments (1^st^-5^th^ flexor digitorum and extensor compartment II/IV/VI of the wrist) were scanned. Machine setting for PD ultrasound was optimized as previously described [25–27].

The severity of ultrasound findings was graded semi-quantitatively on a scale of 0–3 as previously described [25–27]. Each patient’s intra-articular synovitis and tenosynovitis scores (i.e. total GS score and total PD score) were calculated by summing the corresponding GS and PD scores of 11 joints and 8 tendons/tendon compartments, respectively.

### Statistical analysis

Statistical analysis was performed using IBM SPSS Statistics version 22 (IBM Japan, Tokyo, Japan). Normally distributed continuous data were summarized with mean and standard deviation (SD). Non-normally distributed data were summarized with median and interquartile range (IQR) and were analyzed using Spearman’s correlation coefficient. Categorical data were summarized with number and percentage. Multivariate linear regression analysis was performed using forced entry method. Two-sided *P* values less than 0.05 were considered significant.

## Results

### Patients’ characteristics and disease activity

A total of 76 patients fulfilling the inclusion/exclusion criteria were enrolled. These patients were predominated by women (78.9%) with a mean age of 58.4 (SD 14.6) years and a median disease duration of 24 (IQR 8–63.75) months. Rheumatoid factor (RF) and anti-citrullinated protein antibody (ACPA) had been positive in 84.2% and 77.6%, respectively (Table 1).

**Table 1:** Patients’ characteristics, current treatment, and disease activity.

Patients’ characteristics		
	Age, mean ± SD years	58.4 ± 14.6
	Female, n (%)	60 (78.9)
	Disease duration, median (IQR) months	24 (8–63.75)
	RF positive, n (%)	64 (84.2)
	ACPA positive, n (%)	59 (77.6)
	Concomitant autoimmune disease, n (%)	11 (14.5)
Current treatment		
	Treatment naïve, n (%)	17 (22.4)
	Methotrexate, n (%)	39 (51.3)
	Dose, median (IQR) mg/week	10 (8–12)
	Salazosulfapyridine (Sulfasalazine), n (%)	19 (25.0)
	Bucillamine, n (%)	4 (5.3)
	Tacrolimus, n (%)	3 (3.9)
	Auranofin, n (%)	1 (1.3)
	TNF antagonist, n (%)	8 (10.5)
	Tocilizumab, n (%)	4 (5.3)
	Abatacept, n (%)	3 (3.9)
	Corticosteroid, n (%)	22 (28.9)
	Dose, median (IQR) mg/day (prednisolone equivalent)	4.5 (2.375–5)
	NSAID, n (%)	42 (55.3)
Disease activity		
	Swollen joint count/ 28, median (IQR)	2.5 (1–4)
	Tender joint count/ 28, median (IQR)	2 (0–4.75)
	Patient’s global assessment VAS, mean ± SD mm	39.4 ± 23.1
	Physician’s global assessment VAS, mean ± SD mm	36.3 ± 22.8
	Serum C-reactive protein (CRP) level, median (IQR) mg/dL	0.3 (0.0–1.1)
	DAS28-CRP, median (IQR)	3.1 (2.1–4.2)
	SDAI, median (IQR)	12.8 (7–20.675)

SD, standard deviation; IQR, interquartile range; RF, rheumatoid factor; ACPA, anti-citrullinated protein antibody; DMARD, disease modifying anti-rheumatic drug; TNF, tumor necrosis factor; VAS, visual analogue scale; DAS28, Disease Activity Score 28; SDAI, Simplified Disease Activity Index

Nineteen patients (25.0%) underwent ultrasonography for diagnosis and 17 (22.4%) were treatment-naïve. Fifty-seven patients (75.0%) underwent ultrasonography for the evaluation of disease activity. Thirty-nine patients (51.3%) were receiving methotrexate with a median dose of 10 mg/week, 19 (25.0%) salazosulfapyridine, and 15 (19.7%) biologics (TNF antagonists 8, tocilizumab 4, and abatacept 3). Twenty-two patients (28.9%) were receiving corticosteroids with a median dose of prednisolone 4.5 mg/day and 42 (55.3%) non-steroidal anti-inflammatory drugs (NSAIDs) (Table 2).

**Table 2:** Local clinical manifestations and ultrasound scores in dominant hand.

Joint count		
	Swollen joint count/ 11, median (IQR)	1 (1–3)
	Tender joint count/ 11, median (IQR)	1 (0–2)
Morning stiffness		
	Duration, median (IQR) minutes	30 (5–83.75)
	Severity VAS on wakening, median (IQR) mm	43 (5–60)
	Severity VAS at 1/4, median (IQR) mm	5 (0–26.75)
	Severity VAS at 1/2, median (IQR) mm	0 (0–13.75)
	Severity VAS at bedtime, median (IQR) mm	0 (0–5)
Ultrasound		
	Intra-articular synovitis	
	Gray-scale score, median (IQR)	3 (1–6)
	Power Doppler score, median (IQR)	1 (0–4)
	Tenosynovitis	
	Gray-scale score, median (IQR)	1 (0–4)
	Power Doppler score, median (IQR)	0 (0–2)

IQR, interquartile range; VAS, visual analogue scale

Median DAS28 and median SDAI were 3.1 (IQR 2.1–4.2) and 12.8 (7–20.675), respectively (Table 1).

### Clinical and sonographic findings in dominant hand

Clinical and sonographic findings in the dominant hand are summarized in Table 2. Both swollen/tender joint counts and sonographic scores were low, which was consistent with median DAS28 and SDAI values. Median duration of morning stiffness in the same hand was 30 (IQR 5–83.75) minutes. While median severity VAS of hand stiffness on wakening was 43 (IQR 5–60) mm, the majority of patients report disappearance of morning stiffness within the first half of total awake time (Table 2).

**Fig 2:**
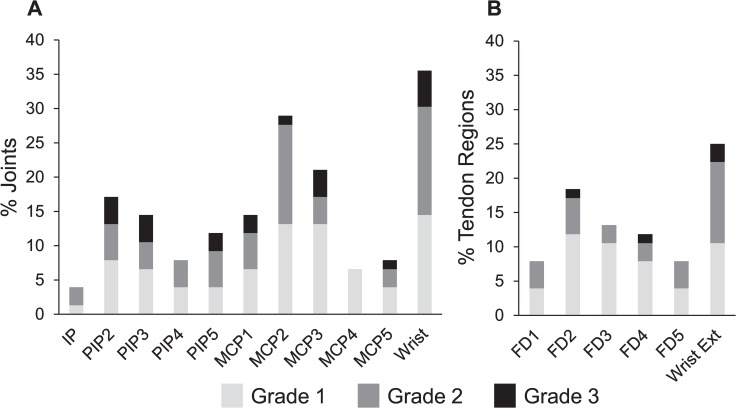
Prevalence of power Doppler signals in each joint/tendon region. Prevalence of each power Doppler (PD) grade > 0 in each joint (A) and each tendon region (B). IP, interphalangeal joint; PIP, proximal interphalangeal joint; MCP, metacarpo-phalangeal joint; FD, flexor digitorum; Wrist Ext, wrist extensor tendons.

Fig 2 shows the prevalence of each PD grade > 0 in each joint and each tendon region. Although there was some similarity in the pattern of distribution between intra-articular synovitis (Fig 2A) and tenosynovitis (Fig 2B), these two types of synovial inflammation did not always coexist in the same anatomical area (agreement 50–63%, κ value 0.200–0.388 [data not shown]).

### Associations between clinical and sonographic findings

We first examined the associations between clinical findings in the dominant hand and total PD scores in the same hand by univariate analysis (Table 3). For intra-articular synovitis, swollen/tender joint counts more strongly correlated with total PD scores (ρ = 0.379–0.561, p ≤ 0.001) than did any parameters of morning stiffness (ρ = 0.217–0.314, p = 0.006–0.021). For tenosynovitis, however, the severity VAS on awakening and the improvement afterwards more strongly correlated with total PD scores (ρ = 0.503–0.561, p < 0.001) than did swollen/tender joint counts (ρ = 0.276–0.388, p = 0.001–0.016).

**Table 3:** Correlations between clinical manifestations and ultrasound scores in dominant hand.

		Joint count		Morning stiffness				
		Swollen joint	Tender joint	Duration	Severity VAS on awakening	Improvement of VAS at 1/4	Improvement of VAS at 1/2	Improvement of VAS at bedtime
Intra-articular synovial power Doppler score								
	ρ^*^	0.561	0.379	0.265	0.314	0.217	0.266	0.306
	p value ^*^	< 0.001	0.001	0.021	0.006	0.060	0.020	0.007
Tenosynovial power Doppler score								
	ρ^*^	0.388	0.276	0.280	0.503	0.505	0.561	0.538
	p value ^*^	0.001	0.016	0.014	< 0.001	< 0.001	< 0.001	< 0.001

^*^ Spearman’s correlation coefficient

VAS, visual analogue scale

We next examined whether the parameters of morning stiffness independently associate with total PD scores by multivariate analysis (Table 4). We tested linear regression models which incorporated swollen/tender joint counts and either one of the 5 parameters on morning stiffness as explanatory variables. For intra-articular synovitis, none of the 5 models retained morning stiffness as a variable which independently associated with total PD score. For tenosynovitis, however, 4 out of the 5 models retained morning stiffness as a single independent variable that significantly associated with total PD score. The duration was the only parameter on morning stiffness that was not identified as a significant independent factor in multivariate analysis.

**Table 4:** Multivariate linear regression models to identify factors which independently associate with power Doppler scores.

	Model 1			Model 2			Model 3			Model 4			Model 5		
	MS: duration			MS: severity VAS on awakening			MS: VAS improvement at first 1/4 of awake time			MS: VAS improvement at first 1/2 of awake time			MS: VAS improvement at bedtime		
	SJC	TJC	MS	SJC	TJC	MS	SJC	TJC	MS	SJC	TJC	MS	SJC	TJC	MS
Intra-articular synovial power Doppler score															
β	0.506	0.243	-0.039	0.469	0.240	0.069	0.465	0.250	0.083	0.453	0.247	0.119	0.446	0.244	0.126
p value	< 0.001	0.025	0.659	< 0.001	0.027	0.499	< 0.001	0.021	0.388	< 0.001	0.022	0.213	< 0.001	0.023	0.197
Tenosynovial power Doppler score															
β	0.218	0.210	-0.067	0.018	0.200	0.384	0.057	0.243	0.332	0.028	0.228	0.429	0.013	0.216	0.436
p value	0.105	0.119	0.541	0.896	0.112	0.002	0.680	0.059	0.005	0.832	0.063	< 0.001	0.919	0.078	< 0.001

MS, morning stiffness; VAS, visual analogue scale; SJC, swollen joint count; TJC, tender joint count; β, standardized coefficient

## Discussion

The major strength of this study is that we determined the activity of intra-articular synovitis and tenosynovitis with PD ultrasound, which has been reported to be more accurate than clinical information [24–26]. Our data indicate that morning stiffness is more closely related with tenosynovitis than it is with intra-articular synovitis. Although the association of morning stiffness with tenosynovitis was investigated decades ago [28, 29], the current study is the first to compare its associations between tenosynovitis and intra-articular synovitis using modern ultrasound equipment.

Tenosynovitis has been recognized as a significant and important feature of RA in studies using modern imaging modalities. Tenosynovitis has been shown to be prevalent and responsive to anti-rheumatic treatment [30, 31], associates with functional disability [32], and predicts erosive disease [33]. Because data from these studies and ours show that joint counts are insufficient to reflect tenosynovitis, evaluating morning stiffness, which can better reflect tenosynovitis, is indispensable in the assessment of disease activity and prognosis of RA.

Another advantage of this study is that we devised a method using a patient-reported diagram to capture the time course of morning stiffness. Our data demonstrate that the severity and the diurnal improvement of morning stiffness are more closely related to the activity of synovial inflammation than is its duration. This does not only apply to tenosynovitis but seems to also apply to intra-articular synovitis (Tables 1 and 2). These data are concordant with the long-standing notion that typical inflammatory joint symptoms are the worst on wakening and improve with activities [23]. Our data indicate that the severity and diurnal improvement are more important than the duration when we ask patients about morning stiffness in order to estimate the activity of synovial inflammation. However, the other aspects of stiffness may have direct impact on patients’ experience and independent meaning as a patient-reported outcome [34].

This study has some limitations. Firstly, relatively small sample size did not allow for stratification by background information such as disease duration and activity. Results may have been different in patients with longer disease duration or higher disease activity with a larger number of involved joints. In addition, we cannot exclude the possibility of selection bias although patients’ characteristics were representative of RA patients in our daily practice. Secondly, our data may not be applicable to joints other than hands. However, hands are the region that is most frequently involved in RA and where patients most frequently reports morning stiffness [1], and are likely to reflect pathophysiology typical of RA. Thirdly, we did not assess patients with other diseases that cause morning stiffness. The presence of a disease control group could have clarified the specificity of our findings in RA patients.

In conclusion, our data demonstrate a pathophysiological link between morning stiffness and tenosynovitis and give an insight into the optimal measurement of morning stiffness. Moreover, our data add to the evidence supporting an independent value of evaluating morning stiffness in the management of RA.

## Supporting Information

S1 File Individual data of each participant. (XLSX)
